# 2-(2,5-Dimethyl­phen­oxy)ethanol

**DOI:** 10.1107/S1600536813005187

**Published:** 2013-02-28

**Authors:** Miho Ukai, Tsunehisa Okuno

**Affiliations:** a Department of Material Science and Chemistry, Wakayama University, Sakaedani, Wakayama, 640-8510, Japan

## Abstract

There are two independent mol­ecules in the asymmetric unit of the title phen­oxy­ethanol derivative, C_10_H_14_O_2_, Each molecule has an approximately planar structure except for the hy­droxy groups (r.m.s. deviations = 0.0281 and 0.0144 Å). The ethyl­enedi­oxy groups have a *gauche* conformation. In the crystal, the mol­ecules form O—H⋯O hydrogen-bonded chains along the *a* axis.

## Related literature

For related structures of phen­oxy­ethanol derivatives, see: Sanyal & Lahti (2006 ▶); Sierra & Lahti (2004 ▶).
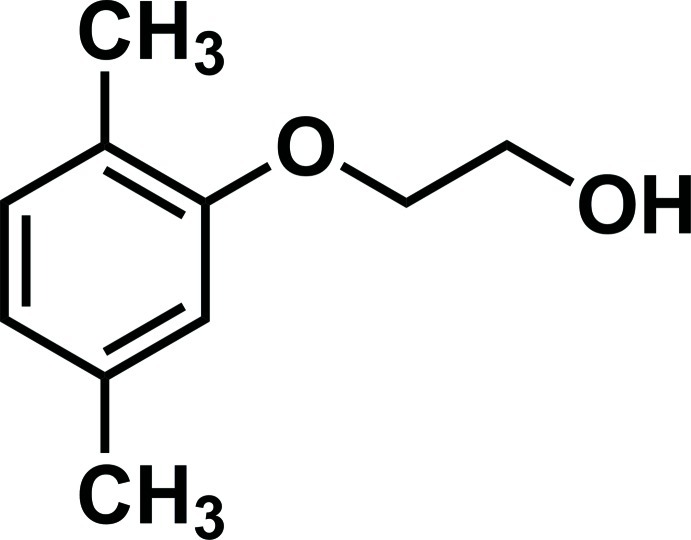


## Experimental

### 

#### Crystal data

C_10_H_14_O_2_
*M* *_r_* = 166.22
Orthorhombic, 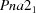

*a* = 8.274 (3) Å
*b* = 13.745 (4) Å
*c* = 17.113 (5) Å
*V* = 1946.2 (11) Å^3^
*Z* = 8
Mo *K*α radiation
μ = 0.08 mm^−1^
*T* = 93 K
0.10 × 0.09 × 0.09 mm

#### Data collection

Rigaku Saturn724+ diffractometer
Absorption correction: numerical (*NUMABS*; Rigaku, 1999 ▶) *T*_min_ = 0.989, *T*_max_ = 0.993
16794 measured reflections
2661 independent reflections
2440 reflections with *F* ^2^ > 2σ(*F* ^2^)
*R*_int_ = 0.027

#### Refinement

*R*[*F* ^2^ > 2σ(*F* ^2^)] = 0.046
*wR*(*F* ^2^) = 0.121
*S* = 1.09
2660 reflections
223 parameters
1 restraint
H-atom parameters constrained
Δρ_max_ = 0.23 e Å^−3^
Δρ_min_ = −0.18 e Å^−3^

### 

Data collection: *CrystalClear* (Rigaku, 2008 ▶); cell refinement: *CrystalClear*; data reduction: *CrystalClear*; program(s) used to solve structure: *SIR92* (Altomare, *et al.*, 1994 ▶); program(s) used to refine structure: *SHELXL97* (Sheldrick, 2008 ▶); molecular graphics: *ORTEP-3 for Windows* (Farrugia, 2012 ▶); software used to prepare material for publication: *CrystalStructure* (Rigaku, 2010 ▶).

## Supplementary Material

Crystal structure: contains datablock(s) global, I. DOI: 10.1107/S1600536813005187/nk2198sup1.cif

Structure factors: contains datablock(s) I. DOI: 10.1107/S1600536813005187/nk2198Isup2.hkl

Supplementary material file. DOI: 10.1107/S1600536813005187/nk2198Isup3.cml

Additional supplementary materials:  crystallographic information; 3D view; checkCIF report

## Figures and Tables

**Table 1:** Hydrogen-bond geometry (Å, °)

*D*—H⋯*A*	*D*—H	H⋯*A*	*D*⋯*A*	*D*—H⋯*A*
O2—H2⋯O4^i^	0.84	1.82	2.623 (3)	159
O4—H4⋯O2^ii^	0.84	1.80	2.626 (3)	167

Symmetry codes: (i) 
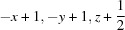
; (ii) 
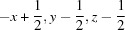
.

**Fig. 1.**
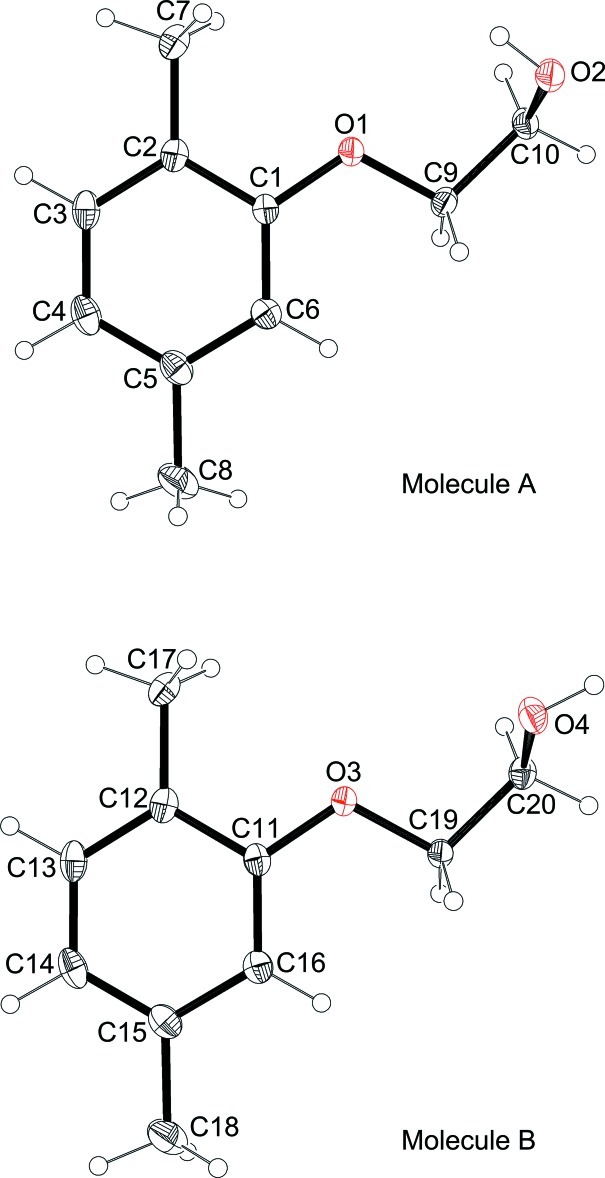
The asymmetric unit of the title compound with atom-numbering scheme. Displacement ellipsoids are drawn at the 50% probability level and H atoms are shown as small spheres.

**Fig. 2.**
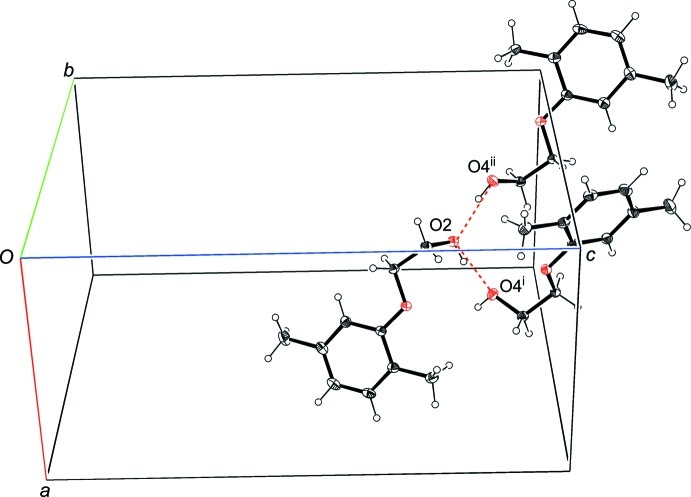
A view of the intermolecular interactions in the title compound. [Symmetry codes: (i) -x + 1, -y + 1, z + 1/2; (ii) -x + 1/2, y + 1/2, z + 1/2.]

## supplementary crystallographic information

### Comment

The title compound, C_10_H_14_O_2_, is a phenoxyethanol derivative whose structures are often observed in phenyleneethynylene derivatives (Sanyal & Lahti, 2006) or phynylenevinylene derivatives (Sierra & Lahti, 2004). Introduction of ethylenedioxy groups was aiming for control of intermolecular interacrions and/or for improvement of solubility to common organic solvents.

The molecular structure of the title compound is shown in Fig. 1. There are two crystallographically independent molecules (A and B) in the unit cell. Both molecules have a planar structure (the C1—C10/O1 plane: r.m.s. deviation = 0.0281 Å, the C11—C20/O3 plane: r.m.s. deviation = 0.0144 Å) except for the hydroxyl groups. The significant difference between A and B is recognized at the torsion angles of ethylenedioxy groups which have a *gauche* conformation.

In the crystal, the molecules form one-dimensional O—H···O hydrogen-bonds (Fig. 2), where the intermolecular distances of O2···O4^i^ and O2···O4^ii^ are 2.623 (3) Å and 2.626 (3) Å, respectively [Symmetry codes: (i) -*x* + 1, -*y* + 1, *z* + 1/2; (ii) -*x* + 1/2, *y* + 1/2, *z* + 1/2.].

### Experimental

The title compound was commercially purchased. Single crystals with sufficient quality were prepared by sublimation at room temperature.

### Refinement

Although CheckCIF suggested the spacegroup *Pbcn*, there existed many reflections which broke systematic absence under *Pbcn* on (0*kl*) reflections. In this reason, the space group *Pna*2_1_ was selected after the lattice transformation. Friedel pairs were merged because the molecule itself was achiral and because there were not any anomalous scattering effects. All H atoms were placed at ideal positions and were refined as riding on their parent atoms. *U*_iso_(H) values of the H atoms were set at 1.2*U*_eq_(parent atom).

### Crystal data

**Table:**

C_10_H_14_O_2_	*F*(000) = 720.00
*M**_r_* = 166.22	*D*_x_ = 1.134 Mg m^−^^3^
Orthorhombic, *P**n**a*2_1_	Mo *K*α radiation, λ = 0.71075 Å
Hall symbol: P 2c -2n	Cell parameters from 6921 reflections
*a* = 8.274 (3) Å	θ = 2.5–31.2°
*b* = 13.745 (4) Å	µ = 0.08 mm^−^^1^
*c* = 17.113 (5) Å	*T* = 93 K
*V* = 1946.2 (11) Å^3^	Block, colourless
*Z* = 8	0.10 × 0.09 × 0.09 mm

### Data collection

**Table:**

Rigaku Saturn724+ diffractometer	2440 reflections with *F*^2^ > 2σ(*F*^2^)
Detector resolution: 28.445 pixels mm^-1^	*R*_int_ = 0.027
ω scans	θ_max_ = 29.0°
Absorption correction: numerical (*NUMABS*; Rigaku, 1999)	*h* = −11→11
*T*_min_ = 0.989, *T*_max_ = 0.993	*k* = −18→14
16794 measured reflections	*l* = −23→21
2661 independent reflections	

### Refinement

**Table:**

Refinement on *F*^2^	Secondary atom site location: difference Fourier map
*R*[*F*^2^ > 2σ(*F*^2^)] = 0.046	Hydrogen site location: inferred from neighbouring sites
*wR*(*F*^2^) = 0.121	H-atom parameters constrained
*S* = 1.09	*w* = 1/[σ^2^(*F*_o_^2^) + (0.0655*P*)^2^ + 0.3857*P*] where *P* = (*F*_o_^2^ + 2*F*_c_^2^)/3
2660 reflections	(Δ/σ)_max_ < 0.001
223 parameters	Δρ_max_ = 0.23 e Å^−^^3^
1 restraint	Δρ_min_ = −0.18 e Å^−^^3^
Primary atom site location: structure-invariant direct methods	

### Special details

**Table:**

Refinement. Refinement was performed using all reflections except for one with very negative *F*^2^. The weighted *R*-factor (*wR*) and goodness of fit (*S*) are based on *F*^2^. *R*-factor (gt) are based on *F*. The threshold expression of *F*^2^ > 2.0 σ(*F*^2^) is used only for calculating *R*-factor (gt).

### Fractional atomic coordinates and isotropic or equivalent isotropic displacement parameters (Å^2^)

**Table:**

	*x*	*y*	*z*	*U*_iso_*/*U*_eq_	
O1	0.83268 (18)	0.62210 (11)	0.70220 (11)	0.0250 (4)	
O2	0.60617 (19)	0.72154 (12)	0.80422 (13)	0.0318 (4)	
O3	0.34067 (18)	0.38176 (11)	0.49535 (10)	0.0250 (4)	
O4	0.13226 (17)	0.28761 (13)	0.39046 (13)	0.0328 (4)	
C1	0.9552 (3)	0.61937 (15)	0.64824 (15)	0.0225 (5)	
C2	1.0979 (3)	0.57326 (15)	0.67334 (17)	0.0257 (5)	
C3	1.2248 (3)	0.5683 (2)	0.62073 (18)	0.0311 (6)	
C4	1.2134 (3)	0.6070 (2)	0.54556 (19)	0.0356 (7)	
C5	1.0719 (3)	0.65250 (17)	0.52115 (19)	0.0328 (6)	
C6	0.9431 (3)	0.65832 (17)	0.57361 (17)	0.0272 (6)	
C7	1.1107 (3)	0.53209 (18)	0.7542 (2)	0.0321 (6)	
C8	1.0567 (4)	0.6947 (3)	0.4399 (2)	0.0479 (8)	
C9	0.6793 (3)	0.65794 (17)	0.67692 (18)	0.0262 (5)	
C10	0.5661 (3)	0.65268 (19)	0.74576 (17)	0.0279 (6)	
C11	0.4644 (3)	0.38227 (16)	0.54812 (16)	0.0241 (5)	
C12	0.6078 (3)	0.42751 (15)	0.52298 (18)	0.0267 (6)	
C13	0.7366 (3)	0.4303 (2)	0.57477 (19)	0.0319 (6)	
C14	0.7263 (3)	0.3917 (2)	0.64977 (18)	0.0345 (7)	
C15	0.5848 (3)	0.34733 (18)	0.67431 (19)	0.0322 (6)	
C16	0.4525 (3)	0.34246 (17)	0.62299 (17)	0.0279 (6)	
C17	0.6194 (3)	0.46858 (18)	0.4415 (2)	0.0321 (6)	
C18	0.5700 (4)	0.3044 (3)	0.7549 (2)	0.0481 (8)	
C19	0.1892 (3)	0.34104 (16)	0.51982 (16)	0.0236 (5)	
C20	0.0756 (3)	0.34828 (17)	0.45185 (17)	0.0264 (5)	
H2	0.6922	0.7046	0.8265	0.0382*	
H3	1.3225	0.5377	0.6363	0.0374*	
H4	0.0557	0.2749	0.3599	0.0393*	
H4A	1.3028	0.6022	0.5109	0.0428*	
H6	0.8457	0.6894	0.5580	0.0327*	
H7A	1.1032	0.5849	0.7925	0.0385*	
H7B	1.2146	0.4987	0.7601	0.0385*	
H7C	1.0225	0.4857	0.7629	0.0385*	
H8A	1.1273	0.6588	0.4041	0.0575*	
H8B	1.0886	0.7633	0.4408	0.0575*	
H8C	0.9444	0.6892	0.4223	0.0575*	
H9A	0.6375	0.6178	0.6333	0.0314*	
H9B	0.6894	0.7260	0.6586	0.0314*	
H10A	0.4542	0.6646	0.7277	0.0335*	
H10B	0.5703	0.5865	0.7685	0.0335*	
H13	0.8351	0.4595	0.5585	0.0383*	
H14	0.8163	0.3958	0.6841	0.0413*	
H16	0.3550	0.3121	0.6392	0.0335*	
H17A	0.6189	0.4153	0.4035	0.0385*	
H17B	0.7199	0.5057	0.4363	0.0385*	
H17C	0.5270	0.5115	0.4317	0.0385*	
H18A	0.6779	0.2962	0.7775	0.0577*	
H18B	0.5163	0.2410	0.7516	0.0577*	
H18C	0.5061	0.3481	0.7879	0.0577*	
H19A	0.1459	0.3776	0.5651	0.0284*	
H19B	0.2035	0.2722	0.5353	0.0284*	
H20A	−0.0341	0.3276	0.4680	0.0317*	
H20B	0.0695	0.4165	0.4336	0.0317*	

### Atomic displacement parameters (Å^2^)

**Table:**

	*U*^11^	*U*^22^	*U*^33^	*U*^12^	*U*^13^	*U*^23^
O1	0.0203 (7)	0.0299 (9)	0.0248 (10)	0.0023 (6)	0.0004 (7)	−0.0004 (7)
O2	0.0259 (7)	0.0418 (9)	0.0278 (10)	0.0065 (6)	−0.0010 (7)	−0.0035 (8)
O3	0.0197 (7)	0.0298 (9)	0.0255 (10)	−0.0032 (6)	−0.0002 (7)	0.0008 (7)
O4	0.0231 (7)	0.0476 (9)	0.0276 (10)	−0.0026 (7)	−0.0004 (7)	−0.0118 (9)
C1	0.0204 (9)	0.0213 (10)	0.0258 (13)	−0.0014 (7)	0.0004 (9)	−0.0039 (10)
C2	0.0240 (9)	0.0219 (10)	0.0312 (15)	0.0004 (8)	−0.0018 (9)	−0.0068 (10)
C3	0.0251 (10)	0.0263 (13)	0.0420 (17)	0.0029 (8)	0.0022 (11)	−0.0107 (11)
C4	0.0323 (11)	0.0323 (13)	0.0423 (18)	−0.0009 (10)	0.0110 (12)	−0.0112 (13)
C5	0.0366 (12)	0.0295 (12)	0.0323 (16)	−0.0020 (9)	0.0082 (12)	−0.0057 (11)
C6	0.0288 (10)	0.0267 (12)	0.0262 (14)	−0.0014 (8)	0.0005 (10)	−0.0039 (10)
C7	0.0285 (11)	0.0309 (12)	0.0369 (18)	0.0037 (9)	−0.0062 (10)	−0.0037 (12)
C8	0.0570 (17)	0.0544 (17)	0.0323 (18)	−0.0001 (14)	0.0147 (14)	−0.0006 (16)
C9	0.0222 (9)	0.0287 (11)	0.0276 (14)	0.0041 (8)	−0.0029 (9)	−0.0007 (11)
C10	0.0207 (10)	0.0351 (13)	0.0280 (15)	0.0009 (8)	0.0021 (9)	−0.0022 (11)
C11	0.0219 (9)	0.0235 (11)	0.0269 (13)	0.0012 (8)	−0.0015 (9)	−0.0065 (10)
C12	0.0239 (9)	0.0215 (10)	0.0347 (16)	−0.0006 (8)	0.0022 (9)	−0.0066 (10)
C13	0.0223 (10)	0.0278 (13)	0.0456 (18)	−0.0028 (8)	−0.0017 (11)	−0.0117 (12)
C14	0.0300 (11)	0.0321 (13)	0.0413 (18)	0.0014 (9)	−0.0131 (11)	−0.0105 (12)
C15	0.0343 (12)	0.0325 (12)	0.0297 (16)	0.0036 (9)	−0.0060 (12)	−0.0043 (12)
C16	0.0260 (10)	0.0281 (12)	0.0295 (15)	0.0016 (8)	−0.0010 (10)	−0.0032 (10)
C17	0.0292 (11)	0.0315 (12)	0.0355 (17)	−0.0056 (9)	0.0072 (10)	0.0009 (12)
C18	0.0497 (16)	0.0611 (19)	0.0335 (19)	0.0021 (14)	−0.0109 (14)	0.0048 (16)
C19	0.0200 (9)	0.0276 (11)	0.0233 (13)	−0.0028 (8)	−0.0001 (8)	−0.0010 (10)
C20	0.0207 (9)	0.0280 (12)	0.0305 (15)	−0.0001 (8)	0.0009 (9)	−0.0029 (11)

### Geometric parameters (Å, º)

**Table:**

O1—C1	1.372 (3)	C3—H3	0.950
O1—C9	1.429 (3)	C4—H4A	0.950
O2—C10	1.417 (4)	C6—H6	0.950
O3—C11	1.365 (3)	C7—H7A	0.980
O3—C19	1.435 (3)	C7—H7B	0.980
O4—C20	1.421 (4)	C7—H7C	0.980
C1—C2	1.407 (3)	C8—H8A	0.980
C1—C6	1.388 (4)	C8—H8B	0.980
C2—C3	1.384 (4)	C8—H8C	0.980
C2—C7	1.499 (5)	C9—H9A	0.990
C3—C4	1.395 (5)	C9—H9B	0.990
C4—C5	1.392 (4)	C10—H10A	0.990
C5—C6	1.396 (4)	C10—H10B	0.990
C5—C8	1.511 (5)	C13—H13	0.950
C9—C10	1.506 (4)	C14—H14	0.950
C11—C12	1.407 (4)	C16—H16	0.950
C11—C16	1.397 (4)	C17—H17A	0.980
C12—C13	1.387 (4)	C17—H17B	0.980
C12—C17	1.507 (5)	C17—H17C	0.980
C13—C14	1.391 (5)	C18—H18A	0.980
C14—C15	1.385 (4)	C18—H18B	0.980
C15—C16	1.405 (4)	C18—H18C	0.980
C15—C18	1.504 (5)	C19—H19A	0.990
C19—C20	1.499 (4)	C19—H19B	0.990
O2—H2	0.840	C20—H20A	0.990
O4—H4	0.840	C20—H20B	0.990
			
O1···O2	2.903 (3)	H2···H20A^iv^	3.2293
O1···C7	2.759 (3)	H2···H20B^ii^	3.1657
O3···O4	2.805 (3)	H3···O3^viii^	3.2303
O3···C17	2.755 (3)	H3···C9^viii^	3.4535
C1···C4	2.772 (4)	H3···C10^viii^	3.1731
C2···C5	2.831 (5)	H3···C11^viii^	2.8669
C3···C6	2.759 (4)	H3···C12^viii^	3.4095
C6···C9	2.809 (4)	H3···C15^viii^	3.4613
C11···C14	2.782 (4)	H3···C16^viii^	2.9000
C12···C15	2.821 (5)	H3···C19^viii^	3.5351
C13···C16	2.769 (4)	H3···H9A^viii^	2.8298
C16···C19	2.804 (4)	H3···H9B^i^	3.4509
O1···O2^i^	3.576 (3)	H3···H10A^viii^	2.5836
O1···O4^ii^	3.465 (3)	H3···H10B^viii^	3.1252
O1···C13	3.512 (4)	H3···H16^viii^	3.1130
O1···C14	3.407 (4)	H3···H19A^viii^	2.9097
O2···O1^iii^	3.576 (3)	H4···O1^v^	3.1850
O2···O3^ii^	3.593 (3)	H4···O2^vii^	1.7996
O2···O4^iv^	2.626 (3)	H4···O2^v^	2.9563
O2···O4^ii^	2.623 (3)	H4···C7^v^	3.4937
O2···C7^iii^	3.493 (4)	H4···C10^vii^	2.7662
O2···C20^iv^	3.417 (4)	H4···H2^vii^	2.3376
O3···O2^v^	3.593 (3)	H4···H2^v^	2.1820
O3···C3^vi^	3.479 (4)	H4···H7A^v^	2.6022
O3···C4^vi^	3.381 (4)	H4···H10A^vii^	2.7247
O4···O1^v^	3.465 (3)	H4···H10B^vii^	3.2001
O4···O2^vii^	2.626 (3)	H4···H17A^x^	2.7674
O4···O2^v^	2.623 (3)	H4A···O3^viii^	3.0584
O4···C10^vii^	3.502 (4)	H4A···C11^viii^	3.3670
C1···C13	3.406 (4)	H4A···C12^viii^	3.4901
C3···O3^viii^	3.479 (4)	H4A···C17^viii^	3.4133
C3···C11^viii^	3.466 (4)	H4A···H6^i^	2.9966
C3···C19^viii^	3.582 (4)	H4A···H8B^i^	3.2319
C4···O3^viii^	3.381 (4)	H4A···H8C^i^	3.4481
C6···C13	3.569 (4)	H4A···H9A^viii^	3.4798
C7···O2^i^	3.493 (4)	H4A···H9B^i^	3.5838
C10···O4^iv^	3.502 (4)	H4A···H17C^viii^	2.6142
C11···C3^vi^	3.466 (4)	H4A···H19A^viii^	3.4762
C13···O1	3.512 (4)	H4A···H20B^viii^	3.4628
C13···C1	3.406 (4)	H6···C4^iii^	3.0126
C13···C6	3.569 (4)	H6···C5^iii^	3.2023
C14···O1	3.407 (4)	H6···C8^iii^	3.5130
C19···C3^vi^	3.582 (4)	H6···H4A^iii^	2.9966
C20···O2^vii^	3.417 (4)	H6···H8B^iii^	2.9956
O1···H2	2.6761	H6···H9B^i^	3.5218
O1···H6	2.6371	H6···H13	3.1605
O1···H7A	2.7675	H6···H17B	3.4341
O1···H7C	2.6573	H7A···O2^i^	2.6680
O1···H10A	3.2155	H7A···O4^ii^	3.1101
O1···H10B	2.4978	H7A···C17^xi^	3.5084
O2···H9A	3.2640	H7A···C20^ii^	3.2347
O2···H9B	2.5862	H7A···H2^i^	3.0416
O3···H16	2.6444	H7A···H4^ii^	2.6022
O3···H17A	2.8254	H7A···H9B^i^	3.5382
O3···H17C	2.5963	H7A···H10A^viii^	3.2964
O3···H20A	3.2233	H7A···H17A^xi^	2.9824
O3···H20B	2.5260	H7A···H17B^xi^	3.1225
O4···H19A	3.2360	H7A···H20A^ii^	3.2843
O4···H19B	2.5572	H7A···H20B^ii^	2.8050
C1···H3	3.2463	H7B···C17^xi^	3.4235
C1···H7A	2.7964	H7B···H10A^viii^	3.0714
C1···H7B	3.3202	H7B···H10B^viii^	3.1834
C1···H7C	2.7453	H7B···H16^viii^	3.4946
C1···H9A	2.6409	H7B···H17A^xi^	3.0518
C1···H9B	2.6484	H7B···H17B^xi^	3.0638
C2···H4A	3.2802	H7B···H18C^viii^	3.2140
C2···H6	3.2858	H7C···C14	3.3800
C3···H7A	3.1158	H7C···H8A^xi^	3.3643
C3···H7B	2.5718	H7C···H14	2.5017
C3···H7C	3.1640	H7C···H18B^ix^	3.1223
C4···H6	3.2535	H7C···H20B^ii^	3.3040
C4···H8A	2.6225	H8A···H7C^xii^	3.3643
C4···H8B	2.9832	H8A···H8C^i^	3.3680
C4···H8C	3.2688	H8A···H18A^xii^	2.7695
C5···H3	3.2670	H8A···H18B^xiii^	3.0820
C6···H4A	3.2561	H8A···H18C^xiii^	3.4546
C6···H8A	3.2772	H8A···H20B^viii^	3.4022
C6···H8B	2.9495	H8B···C18^xiii^	3.4879
C6···H8C	2.6249	H8B···H4A^iii^	3.2319
C6···H9A	2.7832	H8B···H6^i^	2.9956
C6···H9B	2.7175	H8B···H8C^i^	3.0322
C7···H3	2.6742	H8B···H17B^i^	3.3558
C8···H4A	2.6893	H8B···H17C^i^	3.1407
C8···H6	2.6717	H8B···H18A^xiii^	3.5878
C9···H2	2.6408	H8B···H18A^xii^	3.4942
C9···H6	2.4947	H8B···H18B^xiii^	3.3665
C11···H13	3.2509	H8B···H18C^xiii^	2.9685
C11···H17A	2.8222	H8C···C8^iii^	3.5956
C11···H17B	3.3184	H8C···C18^xiii^	3.2752
C11···H17C	2.7183	H8C···H4A^iii^	3.4481
C11···H19A	2.6519	H8C···H8A^iii^	3.3680
C11···H19B	2.6451	H8C···H8B^iii^	3.0322
C12···H14	3.2820	H8C···H17B	3.1414
C12···H16	3.2937	H8C···H18A^xiii^	3.0530
C13···H17A	3.0953	H8C···H18B^xiii^	3.0237
C13···H17B	2.5899	H8C···H18C^xiii^	3.1964
C13···H17C	3.2007	H9A···C3^vi^	3.4888
C14···H16	3.2666	H9A···C12	3.2353
C14···H18A	2.5812	H9A···C13	2.8840
C14···H18B	3.2167	H9A···C14	3.2058
C14···H18C	3.0450	H9A···H3^vi^	2.8298
C15···H13	3.2548	H9A···H4A^vi^	3.4798
C16···H14	3.2697	H9A···H13	3.0072
C16···H18A	3.2977	H9A···H14	3.5011
C16···H18B	2.6582	H9B···C1^iii^	2.8820
C16···H18C	2.8584	H9B···C2^iii^	2.8725
C16···H19A	2.7659	H9B···C3^iii^	2.9151
C16···H19B	2.7254	H9B···C4^iii^	3.0086
C17···H13	2.6849	H9B···C5^iii^	3.0444
C18···H14	2.6823	H9B···C6^iii^	2.9663
C18···H16	2.6629	H9B···H3^iii^	3.4509
C19···H4	3.0880	H9B···H4A^iii^	3.5838
C19···H16	2.4931	H9B···H6^iii^	3.5218
H2···H9A	3.5433	H9B···H7A^iii^	3.5382
H2···H9B	2.8882	H9B···H10A^i^	2.9082
H2···H10A	2.6529	H10A···O1^iii^	3.1299
H2···H10B	2.1538	H10A···O2^iii^	3.5292
H3···H4A	2.3282	H10A···O4^iv^	3.3360
H3···H7A	3.2960	H10A···C1^iii^	3.2658
H3···H7B	2.3611	H10A···C2^vi^	3.3360
H3···H7C	3.3715	H10A···C3^vi^	2.9502
H4···H19B	3.2418	H10A···C6^iii^	3.5896
H4···H20A	2.1200	H10A···C7^vi^	3.4061
H4···H20B	2.3226	H10A···C9^iii^	3.4465
H4A···H8A	2.4601	H10A···H2^iii^	3.2850
H4A···H8B	3.0791	H10A···H3^vi^	2.5836
H4A···H8C	3.5383	H10A···H4^iv^	2.7247
H6···H8A	3.5420	H10A···H7A^vi^	3.2964
H6···H8B	3.0161	H10A···H7B^vi^	3.0714
H6···H8C	2.4630	H10A···H9B^iii^	2.9082
H6···H9A	2.3655	H10A···H17A^ii^	3.2593
H6···H9B	2.2106	H10B···C14	3.5999
H9A···H10A	2.3073	H10B···C17^ii^	3.4361
H9A···H10B	2.4173	H10B···H3^vi^	3.1252
H9B···H10A	2.4288	H10B···H4^iv^	3.2001
H9B···H10B	2.8607	H10B···H7B^vi^	3.1834
H13···H14	2.3267	H10B···H17A^ii^	2.7916
H13···H17A	3.2566	H10B···H17C^ii^	3.2042
H13···H17B	2.3840	H10B···H18C	3.3363
H13···H17C	3.4227	H13···O1	3.3226
H14···H18A	2.3954	H13···C1	2.8586
H14···H18B	3.4669	H13···C2	3.3218
H14···H18C	3.1897	H13···C5	3.3588
H16···H18A	3.5759	H13···C6	2.8862
H16···H18B	2.5364	H13···C19^viii^	3.4168
H16···H18C	2.8783	H13···C20^viii^	3.1030
H16···H19A	2.3263	H13···H6	3.1605
H16···H19B	2.2432	H13···H9A	3.0072
H19A···H20A	2.3351	H13···H19A^viii^	2.8099
H19A···H20B	2.3980	H13···H19B^ix^	3.3894
H19B···H20A	2.4028	H13···H20A^viii^	2.6198
H19B···H20B	2.8631	H13···H20B^viii^	2.9460
O1···H4^ii^	3.1850	H14···O1	3.1293
O1···H10A^i^	3.1299	H14···C1	3.3383
O1···H13	3.3226	H14···C2	3.3789
O1···H14	3.1293	H14···C7	3.2990
O2···H2^iii^	3.5932	H14···H7C	2.5017
O2···H4^iv^	1.7996	H14···H9A	3.5011
O2···H4^ii^	2.9563	H14···H16^ix^	2.9757
O2···H7A^iii^	2.6680	H14···H18B^ix^	2.7574
O2···H10A^i^	3.5292	H14···H19A^viii^	3.4131
O2···H17A^ii^	3.1450	H14···H19B^ix^	3.5612
O2···H20A^iv^	3.2142	H16···C14^x^	3.0020
O3···H2^v^	3.1357	H16···C15^x^	3.1872
O3···H3^vi^	3.2303	H16···C18^x^	3.4700
O3···H4A^vi^	3.0584	H16···H3^vi^	3.1130
O3···H20A^ix^	3.0937	H16···H7B^vi^	3.4946
O4···H2^vii^	3.1153	H16···H14^x^	2.9757
O4···H2^v^	1.8223	H16···H18A^x^	3.1566
O4···H7A^v^	3.1101	H16···H18B^x^	3.4759
O4···H10A^vii^	3.3360	H16···H19B^ix^	3.5801
O4···H17A^x^	2.7994	H17A···O2^v^	3.1450
O4···H20A^ix^	3.4476	H17A···O4^ix^	2.7994
C1···H9B^i^	2.8820	H17A···C7^xii^	3.4722
C1···H10A^i^	3.2658	H17A···C10^v^	3.2409
C1···H13	2.8586	H17A···H2^xvi^	3.5447
C1···H14	3.3383	H17A···H2^v^	3.3280
C2···H9B^i^	2.8725	H17A···H4^ix^	2.7674
C2···H10A^viii^	3.3360	H17A···H7A^xii^	2.9824
C2···H13	3.3218	H17A···H7B^xii^	3.0518
C2···H14	3.3789	H17A···H10A^v^	3.2593
C2···H19A^viii^	3.2902	H17A···H10B^v^	2.7916
C3···H9A^viii^	3.4888	H17A···H19B^ix^	3.4953
C3···H9B^i^	2.9151	H17A···H20A^viii^	3.3032
C3···H10A^viii^	2.9502	H17B···C7^xii^	3.4561
C3···H19A^viii^	2.8652	H17B···H6	3.4341
C4···H6^i^	3.0126	H17B···H7A^xii^	3.1225
C4···H9B^i^	3.0086	H17B···H7B^xii^	3.0638
C4···H17C^viii^	3.4999	H17B···H8B^iii^	3.3558
C4···H19A^viii^	3.2203	H17B···H8C	3.1414
C4···H20B^viii^	3.4561	H17B···H20A^viii^	3.2300
C5···H6^i^	3.2023	H17B···H20B^viii^	3.1414
C5···H9B^i^	3.0444	H17C···C4^vi^	3.4999
C5···H13	3.3588	H17C···H4A^vi^	2.6142
C5···H20B^viii^	3.5728	H17C···H8B^iii^	3.1407
C6···H9B^i^	2.9663	H17C···H10B^v^	3.2042
C6···H10A^i^	3.5896	H17C···H18C^v^	3.1396
C6···H13	2.8862	H18A···C8^xi^	3.5442
C7···H4^ii^	3.4937	H18A···C18^ix^	3.5479
C7···H10A^viii^	3.4061	H18A···H8A^xi^	2.7695
C7···H14	3.2990	H18A···H8B^xiv^	3.5878
C7···H17A^xi^	3.4722	H18A···H8B^xi^	3.4942
C7···H17B^xi^	3.4561	H18A···H8C^xiv^	3.0530
C7···H20B^ii^	3.4845	H18A···H16^ix^	3.1566
C8···H6^i^	3.5130	H18A···H18B^ix^	2.8806
C8···H8C^i^	3.5956	H18A···H18C^ix^	3.3671
C8···H18A^xii^	3.5442	H18B···C8^xiv^	3.3407
C8···H18B^xiii^	3.3407	H18B···C14^x^	3.4809
C8···H18C^xiii^	3.3883	H18B···H7C^x^	3.1223
C9···H3^vi^	3.4535	H18B···H8A^xiv^	3.0820
C9···H10A^i^	3.4465	H18B···H8B^xiv^	3.3665
C10···H3^vi^	3.1731	H18B···H8C^xiv^	3.0237
C10···H4^iv^	2.7662	H18B···H14^x^	2.7574
C10···H17A^ii^	3.2409	H18B···H16^ix^	3.4759
C11···H3^vi^	2.8669	H18B···H18A^x^	2.8806
C11···H4A^vi^	3.3670	H18C···C8^xiv^	3.3883
C11···H19B^ix^	2.9103	H18C···H7B^vi^	3.2140
C11···H20A^ix^	3.1938	H18C···H8A^xiv^	3.4546
C12···H3^vi^	3.4095	H18C···H8B^xiv^	2.9685
C12···H4A^vi^	3.4901	H18C···H8C^xiv^	3.1964
C12···H9A	3.2353	H18C···H10B	3.3363
C12···H19B^ix^	2.8647	H18C···H17C^ii^	3.1396
C12···H20A^viii^	3.3992	H18C···H18A^x^	3.3671
C13···H9A	2.8840	H19A···C2^vi^	3.2902
C13···H19A^viii^	3.4670	H19A···C3^vi^	2.8652
C13···H19B^ix^	2.8771	H19A···C4^vi^	3.2203
C13···H20A^viii^	2.9891	H19A···C13^vi^	3.4670
C14···H7C	3.3800	H19A···C16^x^	3.5619
C14···H9A	3.2058	H19A···H3^vi^	2.9097
C14···H10B	3.5999	H19A···H4A^vi^	3.4762
C14···H16^ix^	3.0020	H19A···H13^vi^	2.8099
C14···H18B^ix^	3.4809	H19A···H14^vi^	3.4131
C14···H19B^ix^	2.9908	H19B···C11^x^	2.9103
C15···H3^vi^	3.4613	H19B···C12^x^	2.8647
C15···H16^ix^	3.1872	H19B···C13^x^	2.8771
C15···H19B^ix^	3.0526	H19B···C14^x^	2.9908
C16···H3^vi^	2.9000	H19B···C15^x^	3.0526
C16···H19A^ix^	3.5619	H19B···C16^x^	3.0076
C16···H19B^ix^	3.0076	H19B···H13^x^	3.3894
C16···H20A^ix^	3.5372	H19B···H14^x^	3.5612
C17···H4A^vi^	3.4133	H19B···H16^x^	3.5801
C17···H7A^xii^	3.5084	H19B···H17A^x^	3.4953
C17···H7B^xii^	3.4235	H19B···H20A^ix^	2.8149
C17···H10B^v^	3.4361	H20A···O2^vii^	3.2142
C17···H20A^viii^	3.4900	H20A···O3^x^	3.0937
C18···H8B^xiv^	3.4879	H20A···O4^x^	3.4476
C18···H8C^xiv^	3.2752	H20A···C11^x^	3.1938
C18···H16^ix^	3.4700	H20A···C12^vi^	3.3992
C18···H18A^x^	3.5479	H20A···C13^vi^	2.9891
C19···H2^v^	3.5076	H20A···C16^x^	3.5372
C19···H3^vi^	3.5351	H20A···C17^vi^	3.4900
C19···H13^vi^	3.4168	H20A···C19^x^	3.3764
C19···H20A^ix^	3.3764	H20A···H2^vii^	3.2293
C20···H2^v^	2.9706	H20A···H7A^v^	3.2843
C20···H7A^v^	3.2347	H20A···H13^vi^	2.6198
C20···H13^vi^	3.1030	H20A···H17A^vi^	3.3032
H2···O2^i^	3.5932	H20A···H17B^vi^	3.2300
H2···O3^ii^	3.1357	H20A···H19B^x^	2.8149
H2···O4^iv^	3.1153	H20B···C4^vi^	3.4561
H2···O4^ii^	1.8223	H20B···C5^vi^	3.5728
H2···C19^ii^	3.5076	H20B···C7^v^	3.4845
H2···C20^ii^	2.9706	H20B···H2^v^	3.1657
H2···H4^iv^	2.3376	H20B···H4A^vi^	3.4628
H2···H4^ii^	2.1820	H20B···H7A^v^	2.8050
H2···H7A^iii^	3.0416	H20B···H7C^v^	3.3040
H2···H10A^i^	3.2850	H20B···H8A^vi^	3.4022
H2···H17A^xv^	3.5447	H20B···H13^vi^	2.9460
H2···H17A^ii^	3.3280	H20B···H17B^vi^	3.1414
			
C1—O1—C9	117.5 (2)	C5—C8—H8A	109.470
C11—O3—C19	117.64 (19)	C5—C8—H8B	109.468
O1—C1—C2	115.3 (3)	C5—C8—H8C	109.474
O1—C1—C6	123.7 (2)	H8A—C8—H8B	109.474
C2—C1—C6	121.0 (3)	H8A—C8—H8C	109.469
C1—C2—C3	117.4 (3)	H8B—C8—H8C	109.472
C1—C2—C7	120.7 (3)	O1—C9—H9A	110.232
C3—C2—C7	121.9 (3)	O1—C9—H9B	110.239
C2—C3—C4	122.0 (3)	C10—C9—H9A	110.237
C3—C4—C5	120.3 (3)	C10—C9—H9B	110.237
C4—C5—C6	118.4 (3)	H9A—C9—H9B	108.510
C4—C5—C8	121.2 (3)	O2—C10—H10A	109.205
C6—C5—C8	120.4 (3)	O2—C10—H10B	109.199
C1—C6—C5	121.0 (3)	C9—C10—H10A	109.216
O1—C9—C10	107.4 (3)	C9—C10—H10B	109.218
O2—C10—C9	112.0 (2)	H10A—C10—H10B	107.903
O3—C11—C12	115.6 (3)	C12—C13—H13	118.933
O3—C11—C16	123.5 (2)	C14—C13—H13	118.940
C12—C11—C16	120.9 (3)	C13—C14—H14	120.026
C11—C12—C13	117.7 (3)	C15—C14—H14	120.024
C11—C12—C17	120.2 (3)	C11—C16—H16	120.006
C13—C12—C17	122.1 (3)	C15—C16—H16	120.001
C12—C13—C14	122.1 (3)	C12—C17—H17A	109.476
C13—C14—C15	119.9 (3)	C12—C17—H17B	109.472
C14—C15—C16	119.3 (3)	C12—C17—H17C	109.470
C14—C15—C18	121.3 (3)	H17A—C17—H17B	109.469
C16—C15—C18	119.4 (3)	H17A—C17—H17C	109.473
C11—C16—C15	120.0 (3)	H17B—C17—H17C	109.467
O3—C19—C20	107.2 (2)	C15—C18—H18A	109.471
O4—C20—C19	109.13 (19)	C15—C18—H18B	109.477
C10—O2—H2	109.470	C15—C18—H18C	109.474
C20—O4—H4	109.472	H18A—C18—H18B	109.467
C2—C3—H3	118.987	H18A—C18—H18C	109.469
C4—C3—H3	118.991	H18B—C18—H18C	109.469
C3—C4—H4A	119.849	O3—C19—H19A	110.279
C5—C4—H4A	119.860	O3—C19—H19B	110.282
C1—C6—H6	119.517	C20—C19—H19A	110.281
C5—C6—H6	119.522	C20—C19—H19B	110.283
C2—C7—H7A	109.477	H19A—C19—H19B	108.533
C2—C7—H7B	109.480	O4—C20—H20A	109.852
C2—C7—H7C	109.477	O4—C20—H20B	109.858
H7A—C7—H7B	109.459	C19—C20—H20A	109.852
H7A—C7—H7C	109.465	C19—C20—H20B	109.858
H7B—C7—H7C	109.469	H20A—C20—H20B	108.282
			
C1—O1—C9—C10	179.42 (15)	C4—C5—C6—C1	−0.3 (4)
C9—O1—C1—C2	−172.98 (16)	C8—C5—C6—C1	179.6 (3)
C9—O1—C1—C6	6.8 (3)	O1—C9—C10—O2	69.9 (3)
C11—O3—C19—C20	−179.47 (16)	O3—C11—C12—C13	179.65 (17)
C19—O3—C11—C12	−176.76 (16)	O3—C11—C12—C17	−2.0 (3)
C19—O3—C11—C16	2.3 (3)	O3—C11—C16—C15	−178.99 (18)
O1—C1—C2—C3	179.72 (16)	C12—C11—C16—C15	0.0 (4)
O1—C1—C2—C7	−0.7 (3)	C16—C11—C12—C13	0.6 (4)
O1—C1—C6—C5	−179.49 (18)	C16—C11—C12—C17	178.96 (19)
C2—C1—C6—C5	0.3 (4)	C11—C12—C13—C14	−1.1 (4)
C6—C1—C2—C3	−0.1 (3)	C17—C12—C13—C14	−179.5 (2)
C6—C1—C2—C7	179.49 (19)	C12—C13—C14—C15	1.0 (4)
C1—C2—C3—C4	−0.1 (4)	C13—C14—C15—C16	−0.4 (4)
C7—C2—C3—C4	−179.7 (2)	C13—C14—C15—C18	179.7 (3)
C2—C3—C4—C5	0.1 (4)	C14—C15—C16—C11	−0.1 (4)
C3—C4—C5—C6	0.1 (4)	C18—C15—C16—C11	179.8 (3)
C3—C4—C5—C8	−179.8 (3)	O3—C19—C20—O4	65.7 (2)

Symmetry codes: (i) *x*+1/2, −*y*+3/2, *z*; (ii) −*x*+1, −*y*+1, *z*+1/2; (iii) *x*−1/2, −*y*+3/2, *z*; (iv) −*x*+1/2, *y*+1/2, *z*+1/2; (v) −*x*+1, −*y*+1, *z*−1/2; (vi) *x*−1, *y*, *z*; (vii) −*x*+1/2, *y*−1/2, *z*−1/2; (viii) *x*+1, *y*, *z*; (ix) *x*+1/2, −*y*+1/2, *z*; (x) *x*−1/2, −*y*+1/2, *z*; (xi) −*x*+2, −*y*+1, *z*+1/2; (xii) −*x*+2, −*y*+1, *z*−1/2; (xiii) −*x*+3/2, *y*+1/2, *z*−1/2; (xiv) −*x*+3/2, *y*−1/2, *z*+1/2; (xv) −*x*+3/2, *y*+1/2, *z*+1/2; (xvi) −*x*+3/2, *y*−1/2, *z*−1/2.

### Hydrogen-bond geometry (Å, º)

**Table:**

*D*—H···*A*	*D*—H	H···*A*	*D*···*A*	*D*—H···*A*
O2—H2···O4^ii^	0.84	1.82	2.623 (3)	159
O4—H4···O2^vii^	0.84	1.80	2.626 (3)	167

Symmetry codes: (ii) −*x*+1, −*y*+1, *z*+1/2; (vii) −*x*+1/2, *y*−1/2, *z*−1/2.

## References

[bb1] Altomare, A., Cascarano, G., Giacovazzo, C., Guagliardi, A., Burla, M. C., Polidori, G. & Camalli, M. (1994). *J. Appl. Cryst.* **27**, 435.

[bb2] Farrugia, L. J. (2012). *J. Appl. Cryst.* **45**, 849–854.

[bb3] Rigaku (1999). *NUMABS* Rigaku Corporation, Tokyo, Japan.

[bb4] Rigaku (2008). *CrystalClear* Rigaku Corporation, Tokyo, Japan.

[bb5] Rigaku (2010). *CrystalStructure* Rigaku Corporation, Tokyo, Japan.

[bb6] Sanyal, N. & Lahti, P. M. (2006). *Cryst. Growth Des.* **6**, 1253–1255.

[bb7] Sheldrick, G. M. (2008). *Acta Cryst.* A**64**, 112–122. 10.1107/S0108767307043930 18156677

[bb8] Sierra, C. A. & Lahti, P. M. (2004). *Chem. Mater.* **16**, 55–61.

